# Follicular Ameloblastoma Presenting As a Sinonasal Tumor

**Published:** 2012-02-01

**Authors:** Sh Shahidi, P Bronoosh, Y Daneshbod

**Affiliations:** 1 Department of Oral and Maxillofacial Radiology, Dental School, Shiraz University of Medical Sciences, Shiraz, Iran; 2 Department of Pathology, Shiraz University of Medical Sciences, Shiraz, Iran

**Keywords:** Follicular ameloblastoma, Sinonasal tract, Differential diagnosis

## Abstract

A case of follicular ameloblastoma of the left maxilla in a 74-year-old man is reported. The tumor was presented as a radiographically solid mass filling the left sinonasal cavity and invaded maxillary alveola. After radical surgery, the patient has pursued a non-aggressive clinical course after 4 years of follow-up. The radiopathological features of this tumor were reviewed and the possibility of its sinonasal epithelium origin was discussed.

## Introduction

Ameloblastoma is a benign epithelial odontogenic tumor, locally invasive and of slow growth.[1] This lesion is believed to exhibit a locally aggressive behaviour with a high level of recurrence, theoretically originated from dental lamina and remains the enamel organ in development and the epithelial cover of odontogenic cysts or from the cells of the basal layer of the oral mucosa.[2]

The estimated incidence of ameloblastomas is approximately 0.5 per million populations per year. There is no distinct gender predilection. Most cases are diagnosed between 30 and 60 years of age.[3] Based on the recent Classification of Odontogenic Tumors, by World Health Organization (WHO), benign ameloblastomas are recognized in four subtypes: the solid/multicystic, the desmoplastic, the unicystic and the extraosseous/peripheral type.[4] Solid ameloblastomas affect the mandible preferably, especially the posterior region with a proportion between the gnathic bones of 1:5.4.[5]

The literature showed that solid ameloblastoma occurred as the least frequent in maxillary bone.[3][5][6] Ameloblastomas may present on conventional radiographs as a unilobular or multilobular corticated radiolucency resembling a cyst. Bony septa may result in a honeycomb appearance. The lesion may remain asymptomatic before a facial swelling develops. Computed tomography (CT) and Magnetic Resonance Images (MRI) may be helpful in establishing the extent of the lesion, particularly when located in the maxilla.[3]

The overwhelming majority of cases affecting the sinonasal cavity are tumors that grow in the maxilla and secondarily extend through the nasal and paranasal cavities. There are only few genuine primary sinonasal ameloblastomas (SNAs) reported without connection with gnathic areas.[7]

The aim of this article was to report a case of follicular ameloblastoma in the maxillary sinus in a 74-year-old man presenting as sinusitis. The clinical features misled a primary care physician into incorrect diagnosis and treatment. Pitfalls of diagnosis and management of ameloblastoma in the maxillary sinus were briefly reviewed.

## Case Report

A 74-year-old man was referred to our department with recurrent sinusitis and a non-healed extraction wound was left after removal of the regional tooth about two years ago. The patient was diagnosed with acute sinusitis by a family doctor. The patient was managed by antibiotic therapy ‘without’ radiographic and histopathological examination.

**Fig. 1:**
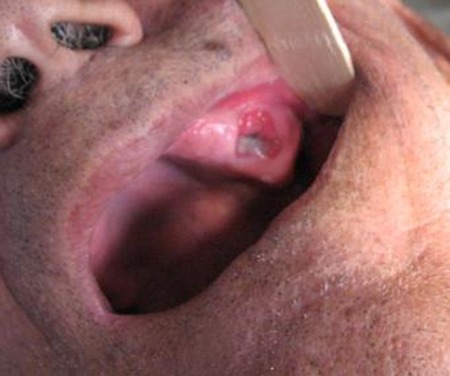
Clinical appearance of the lesion shows extraction socket and alveolar swelling in buccal aspect

The patient’s medical and familial histories were unremarkable. The clinical appearance of the lesion showed a central depressed part with superficial yellowish-white necrotized tissue surrounded by a narrow erythematic rim and mucosal hyperplasia. The lesion was not tender nor bleeding. There was a bony hard alveolar swelling in buccal aspect of premolar and molar region. No significant pus discharge was detected. Mucosal surface was normal in color and texture. Other parts of oral cavity showed normal color and texture. Oroantral fistula had been considered as an early diagnosis (Figure 1).

**Fig. 2:**
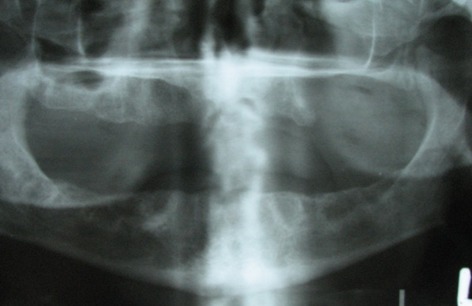
Panoramic view shows complete loss of alveolar process in left premolar and molar region.

**Fig. 3:**
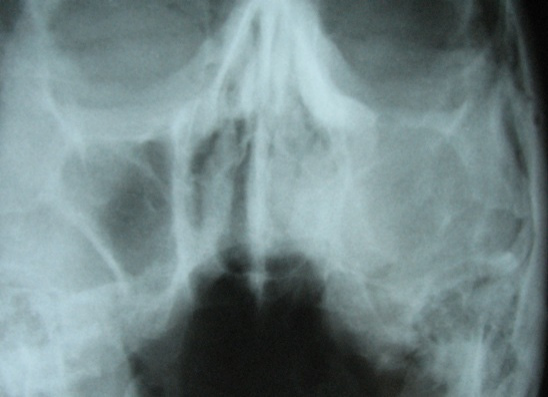
Water`s view shows loss of borders of maxillary sinus and lateral wall of nasal fossa in left side.

On panoramic view, complete loss of alveolar process in left premolar and molar region was detected. Borders of left antrum were not clearly visible. There was no remnant of bone trabequla or sequestra (Figure 2). Water`s view confirmed loss of borders of maxillary sinus and lateral wall of nasal fossa in the left side. Complete haziness of the left antrum and relative haziness of left nasal fossa compared with the right one was obvious (Figure 3).

**Fig. 4:**
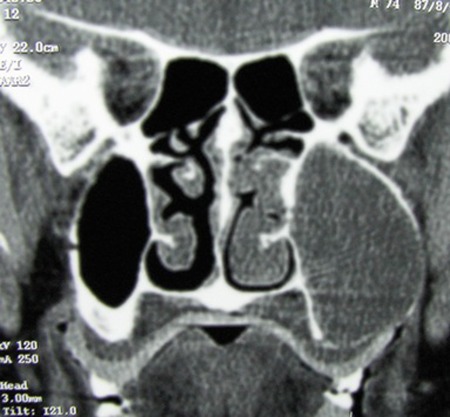
Coronal CT scan view shows a large expansile tumoral lesion located in left maxillary sinus, displacing medial and lateral walls.

**Fig. 5:**
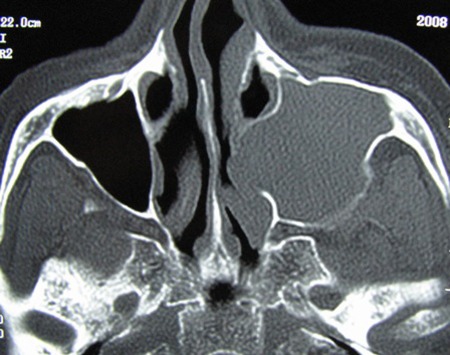
Axial CTscan shows expansile lesion affecting left sinonasal region.

CT demonstrated a massive expansile lesion invading the entire left maxillary sinus. The lateral nasal wall and labial cortex of the maxilla as well as ethmoidal sinuses were involved. Osteomeatal complex and inferior and lateral wall of antrum in the left side were not visible (Figure 4 and Figure 5).

Regarding the size of lesion in antrum, an origin of sinus was suspected.

**Fig. 6:**
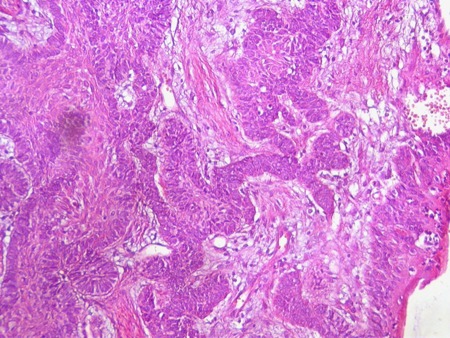
Follicular islands of odontogenic epithelium presenting follicular ameloblastoma

The incisional biopsy revealed fragments of a benign neoplasia of odontogenic origin, characterized by the proliferation of small odontogenic epithelium islands and cords interlarded by a dense fibrous stroma, exhibiting intense collagenized areas. Follicular islands of odontogenic epithelium were bordered by columnar and palisaded ameloblastic cells with polarized hyperchromatic nuclei. Some of the stellate reticulum components of this follicular ameloblastoma showed squamous differentiation (Figure 6).

A radical left maxillectomy was performed. Multiple fragments from left middle and inferior turbinate, left maxillary sinus, left maxilla, left ethmoid tissue and left masseteric region were fixed in formalin submitted to laboratory. Sinonasal follicular ameloblastoma was reported along with mild chronic inflammation in the left ethmoid, maxilla and left middle turbinate tissue. The patient had no sign of recurrence after 4 years follow up.

## Discussion

Ameloblastomas are epithelium-derived odontogenic tumors that typically are originated in jaw bones, primarily involving the mandible and less often the maxilla. The presence of ameloblastomas in the sinonasal region is usually secondary to an extension of a tumor of gnathic origin into this area.[7] However, primary sinonasal ameloblastomas, without extension from the gnathic region, have also been recorded.[3] Previous studies have indicated close relation of the embryological derivation of the sinonasal tract and odontogenic apparatus.[3][8][9] The sinonasal tract and oral cavity communicate until closure of the palatine shelves. This proximity during embryological development could explain the ability of the sinonasal tract mucosa either to incorporate the odontogenic epithelium or to acquire cells capable of odontogenesis during development.[5] It has been suggested that peripheral gnathic and sinonasal ameloblastoma may originate in pluripotential cells of the basal layer of the oral and sinonasal epithelium, respectively.[3][10] The possibility of sinonasal surface epithelium origin of the present case was considered. This direct continuity with the sinonasal surface epithelium could not be proved because there was maxillary bone involvement in this case. Although the development of sinonasal tract ameloblastomas may be initiated after some inductive process on the sinonasal epithelium that results in the neoplastic transformation of retained odontogenic cells and leads to differentiation of ameloblastoma.[3] The presence of chronic inflammation and squamous metaplasia in the specimens taken from left maxillary and ethmoid sinus adjacent to the ameloblastoma could be that initiating factor.

In this case with alveolar process involvement, radiographic views were not enough to make the diagnosis of sinonasal ameloblastoma. In some CT scan slices, primary involvement of maxillary sinus can be suspected. So, it is not easy to exclude extension into maxillary bone from a primary sinonasal tract ameloblastoma. This may support the theory of creating a pathway to maxillary bone through extraction socket. However, the pathologist impression was a sinonasal ameloblastoma.

Some studies claim that, the ameloblastomatous epithelium could have originated in the submucosa and secondarily extended to involve the surface epithelium rather than originating from this epithelium.[3] However, similar to previous studies, we could not find any odonogenic cell source within the submucosa that might represent the source for the development of these tumors.

As we previously noted, most gnathic ameloblastomas appear in patients 35 to 45 years old.[6] By contrast, sinonasal ameloblastomas have a predilection for men of older ages, as in our case. Some studies present the explanation that a longer period of time is required for sinonasal ameloblastomas to reach a large enough size and show some symptoms. Since ameloblastoma is proved to be a locally aggressive tumor a 30 year delay to present symptoms does not seem to be the reason in this case.

The differential diagnosis may remain a challenge in small biopsies if tissue fragments obtained for diagnosis are superficial and the typical histology of the tumor is not well represented in them. CT may aid diagnosis, but MRI seems to be more useful imaging technique, which demonstrates the heterogeneity of the signal, due to its multicystic composition and its irregular contrast uptake, which was not available in this case.[11] According to conventional and advanced radiographic views anthral mucocele and benign aggressive tumoral lesion were considered. Other benign lesions like inverted papilloma, along with ameloblastoma may mimic malignancy by bony erosion. Although malignant lesions such as SCC are in differential diagnosis of maxillary sinus tumoral lesions, there was no evidence of destruction to confirm it. In addition, significant expansion of lesion lowered the possibility of carcinoma.

Surgery is usually the treatment of choice. Recently endoscopic management of benign sinonasal tumors has resulted in less radical surgical approach, decreased morbidity and better tumor control.[12] Hertog et al. proposed annual follow up during five years after radical surgery of solid ameloblastomas.[3] In cases of maxillary involvement, up to a period of at least 10 years follow up has been recommended as these lesions were more dangerous clinically and can invade adjacent sinus and vital structures.[3][11]

Generally, ameloblastomas are originated in jaw bones. Sinonasal ameloblastomas are exceedingly rare tumors without extension from the gnathic region. The prognosis of the treatment is basically dependent to the extension of the lesion and adjacent structures involvement rather than origin of lesion.
